# Structural characterization, thermal properties, and molecular motions near the phase transition in hybrid perovskite [(CH_2_)_3_(NH_3_)_2_]CuCl_4_ crystals: ^1^H, ^13^C, and ^14^N nuclear magnetic resonance

**DOI:** 10.1038/s41598-020-77931-0

**Published:** 2020-11-30

**Authors:** Ae Ran Lim

**Affiliations:** Analytical Laboratory of Advanced Ferroelectric Crystals, Department of Science Eduction, Jeonju University, Jeonju, 55069 Korea

**Keywords:** Chemical physics, Physics, Chemistry, Inorganic chemistry

## Abstract

The structural characterization of the [(CH_2_]_3_(NH_3_)_2_]^+^ cation in the perovskite [(CH_2_)_3_(NH_3_)_2_]CuCl_4_ crystal was performed by solid-state ^1^H nuclear magnetic resonance (NMR) spectroscopy. The ^1^H NMR chemical shifts for NH_3_ changed more significantly with temperature than those for CH_2_. This change in cationic motion is enhanced at the N-end of the organic cation, which is fixed to the inorganic layer by N–H···Cl hydrogen bonds. The ^13^C chemical shifts for CH_2_-1 increase slowly without any anomalous change, while those for CH_2_-2 move abruptly compared to CH_2_-1 with increasing temperature. The four peaks of two groups in the ^14^N NMR spectra, indicating the presence of a ferroelastic multidomain, were reduced to two peaks of one group near T_C2_ (= 333 K); the ^14^N NMR data clearly indicated changes in atomic configuration at this temperature. In addition, ^1^H and ^13^C spin–lattice have shorter relaxation times (T_1ρ_), in the order of milliseconds because T_1ρ_ is inversely proportional to the square of the magnetic moment of paramagnetic ions. The T_1ρ_ values for CH_2_ and NH_3_ protons were almost independent of temperature, but the CH_2_ moiety located in the middle of the N–C–C–C–N bond undergoes tumbling motion according to the Bloembergen–Purcell–Pound theory. Ferroelasticity is the main cause for the phase transition near T_C2_.

## Introduction

The hybrid organic–inorganic compounds, [(CH_2_)_*n*_(NH_3_)_2_]*MX*_4_ (*M* = Mn, Fe, Co, Cu, and Cd, *X* = Cl, Br, *n* = 2, 3 …), are well-known, and have been studied extensively for groups of these crystals. Most of these structures exhibit ferroelastic or ferroelectric phase transitions. The physical properties and phase transitions are related to their structure and the interaction between cationic and anionic sublattices. An interesting family of hybrid compounds is perovskite-type crystals with (CH_2_)_*n*_(NH_3_)_2_ and *MX*_4_-layered metal-halogen anionic sublattice^1–8^. In [(CH_2_)_*n*_(NH_3_)_2_]*MX*_4_, the hydrogen bonds form between the NH_3_ groups at both ends of the aliphatic chains and *X*-atoms of the perovskite-type layer. Hybrid organic–inorganic materials based on the perovskite structures are interesting owing to their potential applications^9–15^. On the one hand, the ferroelastic orientation state in a material is determined by its spontaneous strain tensor, similar to how spontaneous polarization leads to ferroelctricity^16^. Moreover, ferroelasticity is commonly observed in materials with a perovskite crystal structure. Recently, the ferroelastic twin domain observed in hybrid organic–inorganic perovskite has also garnered much attention^17–19^.

Among these materials, [(CH_2_)_3_(NH_3_)_2_]CuCl_4_ [bis (propylene-1, 3-diammonium) tetrachlorocuprate] with *n* = 3 and *M* = Cu undergoes two phase transitions, at temperatures of 333 K (= T_C2_) and 434 K (= T_C1_)^20^.

**Figure 1:**
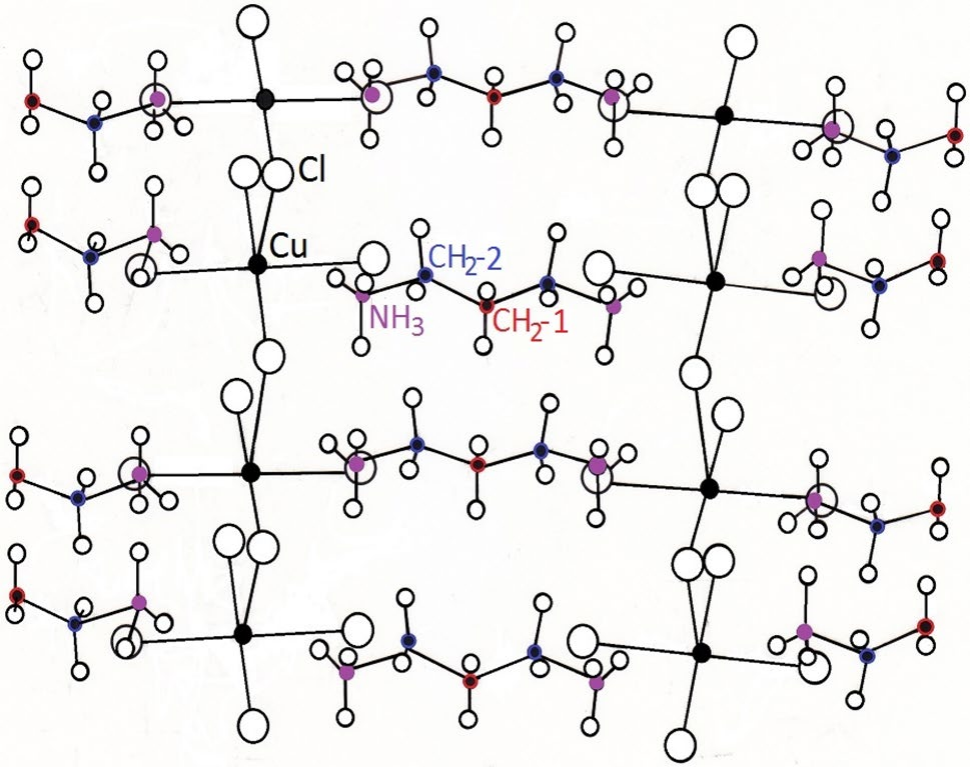
Structural geometry of [(CH_2_)_3_(NH_3_)_2_]CuCl_4_ at room temperature. Here, CH_2_ between CH_2_ and CH_2_ is named CH_2_-1, and CH_2_ close to NH_3_ is named CH_2_-2.

The crystal at room temperature has an orthorhombic structure with a space group *Pnma*. The unit cell dimensions are *a* = 7.202 Å, *b* = 18.260 Å, *c* = 7.515 Å, and Z = 4^21^. The crystal structure consists of chloro-bridged deformed tetragonal (CuCl_4_)^2-^ to form two-dimensional layers. The chlorocuprate sheet is hydrogen bonded to [(CH_2_)_3_(NH_3_)_2_] in alternating layers. The structural geometry of the [(CH_2_)_3_(NH_3_)_2_]CuCl_4_ is shown in Fig. 1^21^. Extensive hydrogen bonding of the N–H···Cl occurs between the Cu–Cl layer and the alkylammonium chain. The organic chains are extended along the *a* direction. The organic chains NH_3_–CH_2_–CH_2_–CH_2_–NH_3_ are almost identical, and the skeleton N–C–C–C–N is planar. Above 434 K, the symmetry is monoclinic with space group *B2/m* and lattice constants *a* = 7.309 Å, *b* = 8.866 Å, *c* = 7.614 Å, α = 95.365°, and Z = 2^22^. The lattice constants *a* and *c* in the monoclinic structure are comparable with those in the room temperature structure, whereas the *b* parameter in the monoclinic structure is half of that in the room temperature structure.

According to the previously reported, the Phelps et al.^21^ and Czupinski et al.^23^ determined the structural phase transition for (CH_2_)_3_(NH_3_)_2_CuCl_4_. And, the structural, dielectric, and conductive properties were discussed by Mostafa et al.^20^. In addition, the structural phase transition was analysed by x-ray and optical studies^22^, where ferroelastic multidomain walls were observed in the orthorhombic phase. Iqbal et al.^1^ reported Raman scattering results at various temperatures above and below the respective magnetic ordering temperature (149 K) and in a magnetic field up to 10 kg. The crystal structure, magnetic and optical properties have been studied by only a few researchers. In addition, the thermal properties, the structural and molecular dynamics of the [(CH_2_)_3_(NH_3_)_2_]CuCl_4_ crystal have not been studied in detail.

Here, differential scanning calorimetry (DSC) and thermogravimetric analysis (TGA) experiments were performed to provide a better understanding of the phase transition temperatures and thermal properties of [(CH_2_)_3_(NH_3_)_2_]CuCl_4_. In addition, the structural characterizations of the [(CH_2_)_3_(NH_3_)_2_] cation were studied in detail by magic angle spinning (MAS) nuclear magnetic resonance (NMR) and static NMR methods. The temperature dependences of the chemical shifts and spin–lattice relaxation times T_1ρ_ were measured by ^1^H MAS NMR and ^13^C cross-polarization (CP)/MAS NMR to highlight the role of the cation in [(CH_2_)_3_(NH_3_)_2_]CuCl_4_. In addition, ^14^N static NMR spectra of [(CH_2_)_3_(NH_3_)_2_]CuCl_4_ single crystals were acquired. Based on these results, the structural characterizations for NH_3_–CH_2_–CH_2_–CH_2_–NH_3_ are discussed as a function of temperature. In particular, the hydrogen bonding of the N–H···Cl between the Cu–Cl layer and the alkylammonium chain within the [(CH_2_)_3_(NH_3_)_2_]CuCl_4_ is expected to give important information regarding the fundamental mechanisms that enable various potential applications.

## Experimental

Crystals of [(CH_2_)_3_(NH_3_)_2_]CuCl_4_ were prepared by mixing equimolar amounts of NH_2_(CH_2_)_3_NH_2_·2HCl and CuCl_2_ (1:1 ratio) in aqueous solution. Then, the resulting mixture was allowed to slowly evaporate at 300 K. The crystals grew as rectangular parallelepipeds, with dimensions of 7 × 7 × 2 mm^3^.

The structure of the [(CH_2_)_3_(NH_3_)_2_]CuCl_4_ crystal at 300 K was analysed using an X-ray diffraction system equipped with a Cu-Kα radiation source at the KBSI, Seoul Western Center. DSC (TA Instruments, DSC 25) was conducted at a heating rate of 10 °C/min from 190 to 600 K under nitrogen gas. TGA was performed using a thermogravimetric analyser (TA Instruments) from 300 to 680 K at the same heating rate. The sample weights used for DSC and TGA experiments were 6.23 and 7.53 mg, respectively. Optical observations were performed using an optical polarized microscope in the temperature range of 300–600 K, where the as-grown crystals were placed on a Linkam THM-600 heating stage.

NMR spectra of [(CH_2_)_3_(NH_3_)_2_]CuCl_4_ crystals were obtained using a 400 MHz Avance II + Bruker solid-state NMR spectrometer, equipped with 4 mm CP/MAS probes (at the KBSI, Seoul Western Center). The Larmor frequencies to ^1^H MAS NMR and ^13^C CP/MAS NMR experiments were at ω_0_/2π = 400.13 and 100.61 MHz, respectively. A MAS rate of 10 kHz was used to minimize the spinning sideband. The NMR chemical shifts were recorded using tetramethylsilane (TMS) as the standard. The T_1ρ_ values were measured using a π/2 − t sequence by changing the spin-locking pulses, and the width of the π/2 pulse was 3.3 μs. The spin-lock power on the ^1^H and ^13^C channel was 75.76 kHz. The ^13^C T_1ρ_ values were obtained by changing the duration of the ^13^C spin-locking pulse applied after the CP preparation period. In addition, ^14^N NMR spectra of a [(CH_2_)_3_(NH_3_)_2_]CuCl_4_ single crystal were measured with a Larmor frequency of 28.90 MHz. The resonance frequency was referenced with respect to NH_3_NO_3_ as a standard sample. The ^14^N NMR experiments were performed using a solid-state echo sequence: 8 μs—tau (16 μs) – 8 μs—tau (16 μs). NMR data could not be obtained because the NMR spectrometer could not operate at temperatures above 430 K. The true temperature at spinning condition of 10 kHz was adjusted based on the sample temperature, suggested by Guan and Stark^24^. The temperature change was maintained within the error range of ± 0.5 K while adjusting nitrogen gas flow and heater current.

## Experimental results

**Figure 2:**
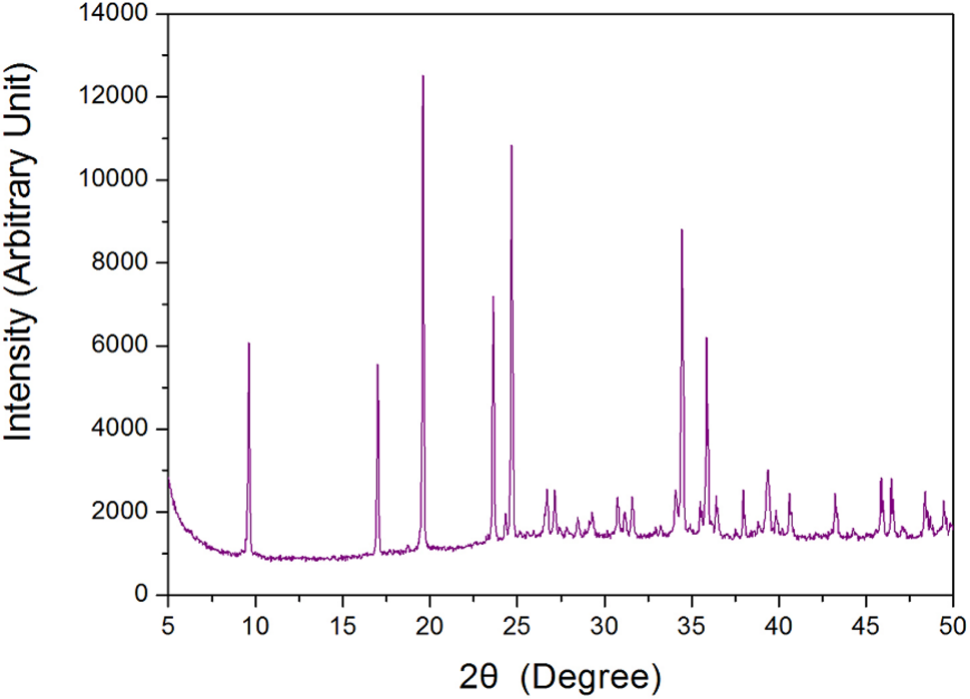
X-ray diffraction pattern of the [(CH_2_)_3_(NH_3_)_2_]CuCl_4_ crystal at 300 K.

**Figure 3:**
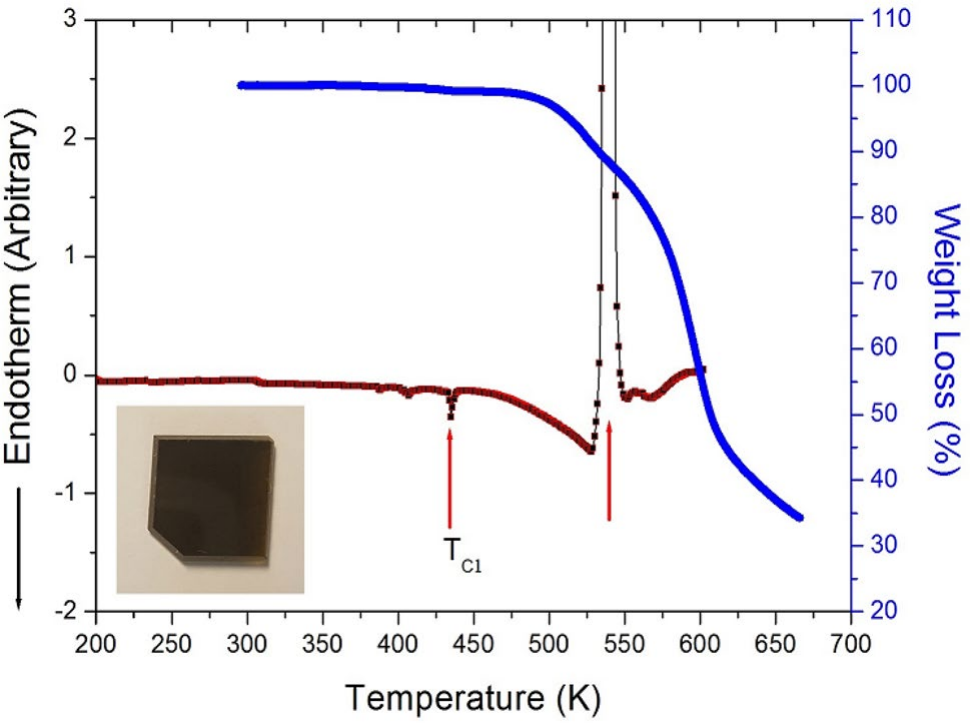
Thermogravimetric analysis (TGA) and differential scanning calorimetry (DSC) thermogram of [(CH_2_)_3_(NH_3_)_2_]CuCl_4_ (Inset: photograph of the crystal at 300 K).

The X-ray powder diffraction pattern of the [(CH_2_)_3_(NH_3_)_2_]CuCl_4_ crystal at room temperature is displayed in Fig. 2, and this result was consistent with that reported by Czapla et al.^23^ The results of the DSC analysis of [(CH_2_)_3_(NH_3_)_2_]CuCl_4_ under a nitrogen atmosphere are shown in Fig. 3. An endothermic peak at 434 K and a exothermic peak at 539 K where observed. However, the peak around 334 K reported previously^20^ was not observed. To confirm that the DSC peaks at 434 K and 539 K were consistent with the structural phase transition, TGA was performed. The measured TGA curves are also shown in Fig. 3. Good thermal stability was observed up to around 480 K; above this temperature, the first signs of weight loss were observed, indicating the onset of partial thermal decomposition. The crystalline structure of the compound [(CH_2_)_3_(NH_3_)_2_]CuCl_4_ (M = 280.49 mg) breaks down at high temperatures. Considering the TGA results and possible chemical reactions, the solid residue amounts were calculated. The weight loss of 13% at around 539 K (see Fig. 3) was likely due to the decomposition of the HCl moieties, which is consistent with the exothermic peak at the same temperature in the DSC curve. The weight sharply decreased between 500 and 600 K, with a corresponding weight loss of 65% around 650 K. This result is consistent with previous TGA data^23^. Further, optical polarizing microscopy was used to understand the crystal’s phase transition, thermal decomposition, and melting mechanism. The color of the crystal was dark brown at room temperature, as illustrated in the inset of Fig. 3. While there were no changes observed from room temperature to 523 K, it began to melt slightly at approximately 539 K. Above 600 K, the crystal emitted an odour, and its surface and edges melted considerably (see Suppplementary Information 1).

**Figure 4:**
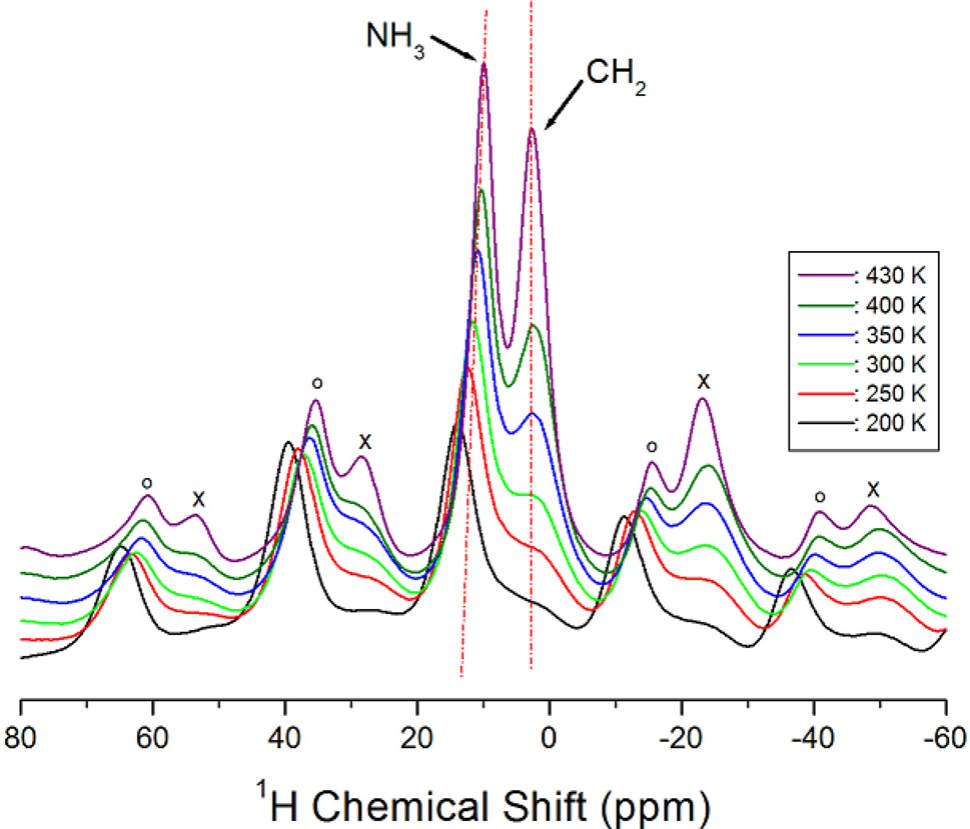
^1^H MAS NMR spectra for CH_2_ and NH_3_ of [(CH_2_)_3_(NH_3_)_2_]CuCl_4_ at various temperatures (spinning sidebands are indicated by cross and open circles).

The chemical shifts of the ^1^H NMR spectrum of [(CH_2_)_3_(NH_3_)_2_]CuCl_4_ crystals were obtained with increasing temperature, as shown in Fig. 4. Two peaks in the NMR spectra are indicated in the figure; the spinning sidebands for CH_2_ are represented with crosses, and those for NH_3_ are marked with open circles. At 300 K, the ^1^H NMR chemical shift for CH_2_ was observed at δ = 2.76 ppm, whereas that for NH_3_ was at δ = 11.48 ppm. Below 300 K, the signal for ^1^H of CH_2_ had very low intensity and could not be easily identified. The ^1^H peak for CH_2_ did not significantly change with increasing temperature, while for NH_3_, the change in the chemical shift was dependent on temperature (see the Supplementary Information 2).

**Figure 5:**
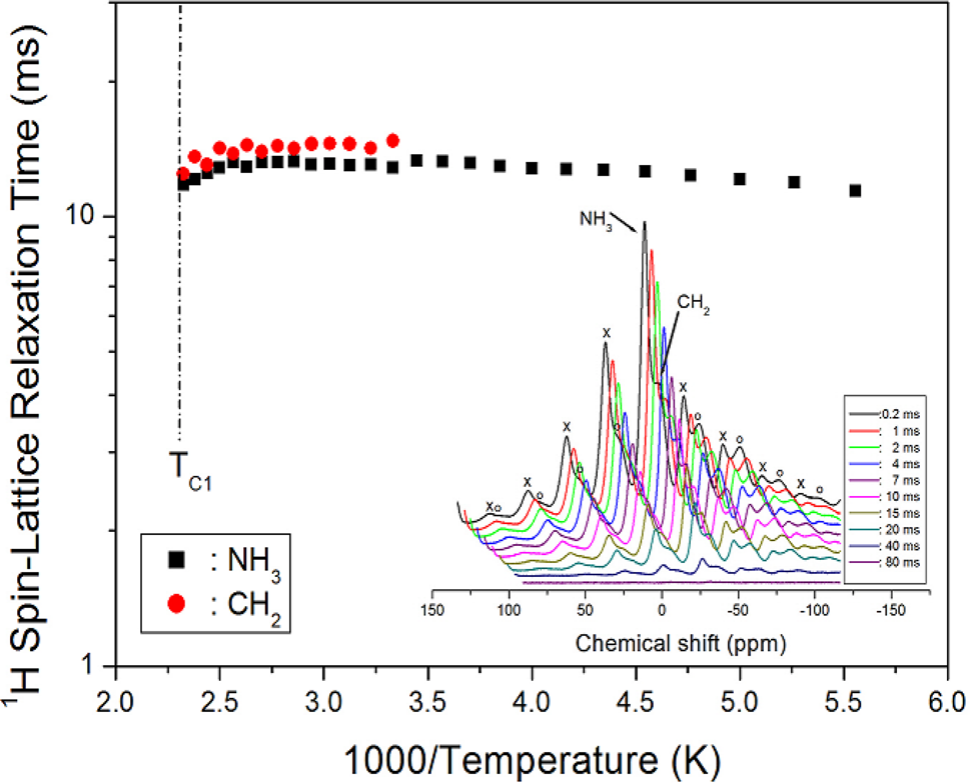
^1^H NMR spin–lattice relaxation times T_1ρ_ for CH_2_ and NH_3_ ions of [(CH_2_)_3_(NH_3_)_2_]CuCl_4_ as a function of inverse temperature (inset: ^1^H NMR spectrum at several delay times at 300 K).

The ^1^H NMR spectra were also measured with several delay times, and the intensity of NMR spectra as a function of delay time followed a single exponential function. The rate of decay of the spin-locked proton magnetization is characterized by T_1ρ_^25–27^:

1 $${\text{I}}\left( t \right) \, = {\text{ I}}(0){\text{exp}}( - t/{\text{T}}_{{1\rho}} ),$$

where I(*t*) and I(0) are the signal intensity at time *t* and *t* = 0, respectively. The ^1^H NMR signals of CH_2_ and NH_3_ measured at 300 K were plotted as a function of delay time over the range of 0.2–80 ms, as shown in the inset of Fig. 5. It can be seen that the ^1^H NMR signal intensities varied with the delay time. From the slope of the intensity vs. delay time curve, ^1^H T_1ρ_ values for [(CH_2_)_3_(NH_3_)_2_]CuCl_4_ were obtained from the CH_2_ and NH_3_ peaks as a function of inverse temperature. Changes in T_1ρ_ values above T_C1_ were not observed outside this temperature because of the limitation of the NMR spectrometer. The ^1^H T_1ρ_ values for CH_2_ and NH_3_ were of the order of 10 ms, and their values were almost independent of temperature (see Fig. 5).

**Figure 6:**
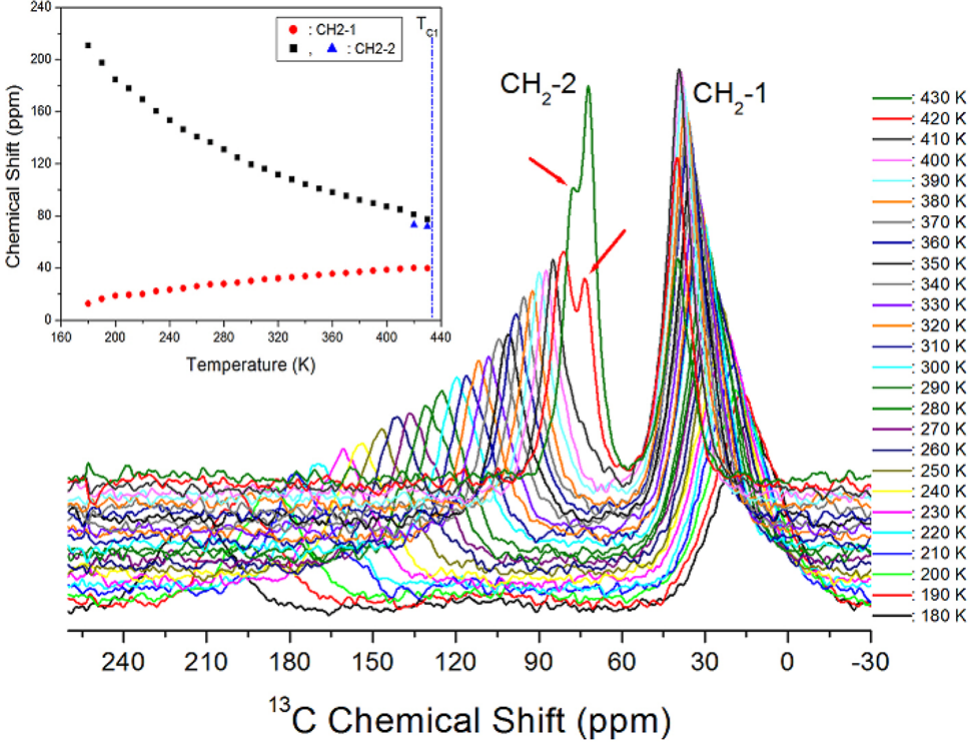
^13^C chemical shifts for CH_2_-1 and CH_2_-2 of (CH_2_)_3_(NH_3_)_2_CuCl_4_ as a function of temperature.

The ^13^C NMR chemical shifts for CH_2_ in [(CH_2_)_3_(NH_3_)_2_]CuCl_4_ were measured as a function of temperature, as shown in Fig. 6. At all temperatures, the ^13^C MAS NMR spectra showed two resonance signals. The ^13^C MAS NMR spectrum for TMS was observed at 38.3 ppm at 300 K, which was used to calibrate the device to 0 ppm for determining the chemical shift in ^13^C^28^. Here, CH_2_ between CH_2_ and CH_2_ is named CH_2_-1, and CH_2_ close to NH_3_ is named CH_2_-2. At 300 K, the two resonance signals were recorded at chemical shifts of δ = 28.78 and δ = 124.97 ppm for CH_2_-1 and CH_2_-2, respectively. The ^13^C chemical shifts for CH_2_ were different CH_2_-1 far away from NH_3_ and CH_2_-2 close to NH_3_. The small ^13^C resonance peaks indicated by arrow at 420 K and 430 K were attributed to a splitting of the CH_2_-2. The ^13^C chemical shift for CH_2_-1 increased slowly and monotonously without an anomalous change with increasing temperature, whereas those for CH_2_-2 moved abruptly to the lower side with increasing temperature compared to CH_2_-1, as shown in the inset in Fig. 6.

The ^13^C full-width at half-maximum (FWHM) values of the NMR peaks for CH_2_-1 and CH_2_-2 decreased with increasing temperature. Broader line widths are observed for more rigid lattices, where motional narrowing is quenched, as shown by the increase in line widths at lower temperatures. The line widths of ^13^C for CH_2_-1 and CH_2_-2 were the same within experimental uncertainty, where the line width narrowed from 30 to 10 ppm with increasing temperature from 180 to 430 K, respectively (see the Supplementary Information 3).

**Figure 7:**
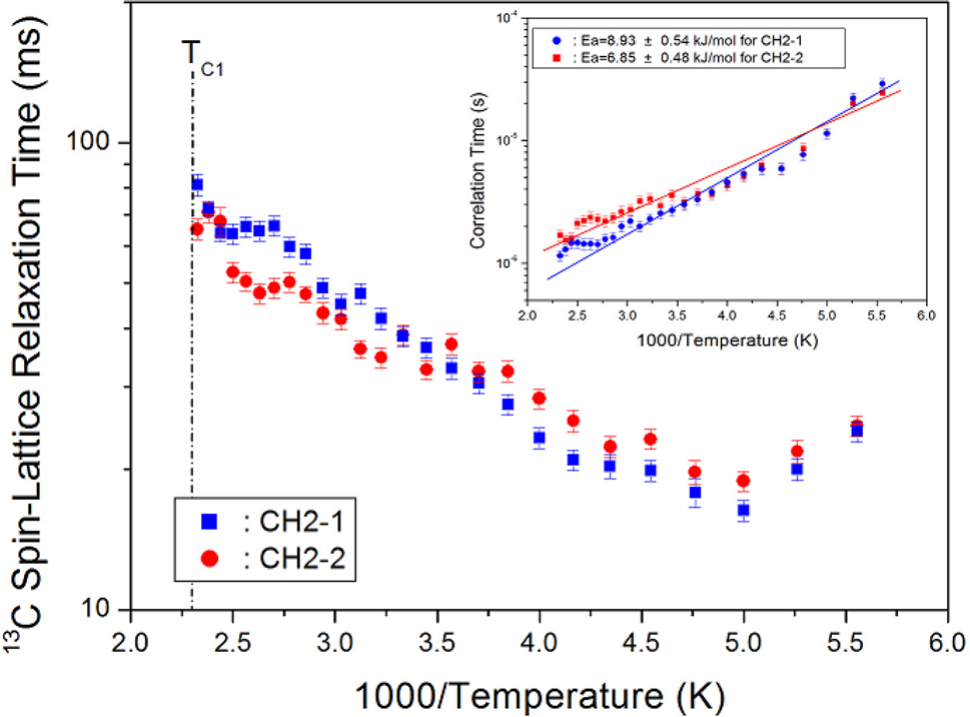
^13^C NMR spin–lattice relaxation times T_1ρ_ for CH_2_-1 and CH_2_-2 of [(CH_2_)_3_(NH_3_)_2_]CuCl_4_ as a function of inverse temperature (Inset: Correlation times for CH_2_-1 and CH_2_-2 in [(CH_2_)_3_(NH_3_)_2_]CuCl_4_ as a function of inverse temperature. Solid lines represent the activation energies).

The integration change of the ^13^C NMR spectra obtained by increasing the delay time was measured. All decay curves for CH_2_-1 and CH_2_-2 were described by a single exponential function, as shown by Eq. (1). ^13^C T_1ρ_ values were measured by the spin-locking pulse sequence with a locking pulse of 75.76 kHz. From the slope of their recovery traces, the ^13^C T_1ρ_ values were obtained for the CH_2_-1 and CH_2_-2 as a function of 1000/temperature, as shown in Fig. 7. Although no change in T_1ρ_ values was observed near T_C2_, T_1ρ_ values measured for 180–430 K indicated a much slower dynamics of carbon motion. The T_1ρ_ vs. temperature curve showed minima of 16.32 and 18.87 ms for CH_2_-1 and CH_2_-2 at 200 K, respectively. This trend indicates that distinct molecular motions exist, where the minimum T_1ρ_ was attributed to the uniaxial rotation of CH_2_ ions. The T_1ρ_ values were described by the correlation time τ_C_ for molecular motion, based on the theory of Bloembergen–Purcell–Pound (BPP). The T_1ρ_ value for the molecular motion is given by^27,29^:

2 $${\text{T}}_{{1\rho}}^{{ - {1}}} = {\text{ C }}(\gamma_{{\text{I}}}^{{2}} \gamma_{{\text{e}}}^{{2}} \mu_{{\text{B}}}^{{2}} {\text{S}}\left( {{\text{S}} + {1}} \right)/r^{{6}} )\left[ {{4}f_{{\text{a}}} + f_{{\text{b}}} + { 3}f_{{\text{c}}} + { 6}f_{{\text{d}}} + { 6}f_{{\text{e}}} } \right]$$

where *f*_a_ = τ_C_ /[1 + ω_1_^2^τ_C_^2^], *f*_b_ = τ_C_ /[1 + (ω_C_ ‒ ω_e_)^2^τ_C_^2^], *f*_c_ = τ_C_ /[1 + ω_C_^2^τ_C_^2^], *f*_d_ = τ_C_ /[1 + (ω_C_ + ω_e_)^2^τ_C_^2^], and *f*_e_ = τ_C_ /[1 + ω_e_^2^τ_C_^2^]. Here, C is a coefficient, γ_e_ is the gyromagnetic ratio of the electron, S is the spin number of the paramagnetic ion, *r* is the distance between the paramagnetic ion and the carbon, ω_e_ is the Larmor frequency of electron, and ω_1_ is the spin-lock field. When ω_C_τ_C_ = 1, T_1ρ_ is at its minimum, so a relationship between T_1ρ_ and ω_1_ was applied to obtain the coefficient in Eq. (2). Using this coefficient, we calculated τ_C_ as a function of temperature. According to BPP theory, the local field fluctuation is governed by the thermal motion of CH_2_-1 and CH_2_-2, which is activated by thermal energy. In this case, τ_C_ is described by Arrhenius behaviour: τ_C_ = τ_o_exp(‒E_a_/k_B_T), where τ_o_, E_a_, and k_B_ are the pre-correlation time, activation energy of the motions, and Boltzmann constant, respectively^27^. As the magnitude of E_a_ depends on the molecular dynamics, we plotted τ_C_ vs. 1000/T on a logarithmic scale (inset of Fig. 7), which gave E_a_ values for CH_2_-1 and CH_2_-2 of 8.93 ± 0.54 and 6.85 ± 0.48 kJ/mol, respectively.

**Figure 8:**
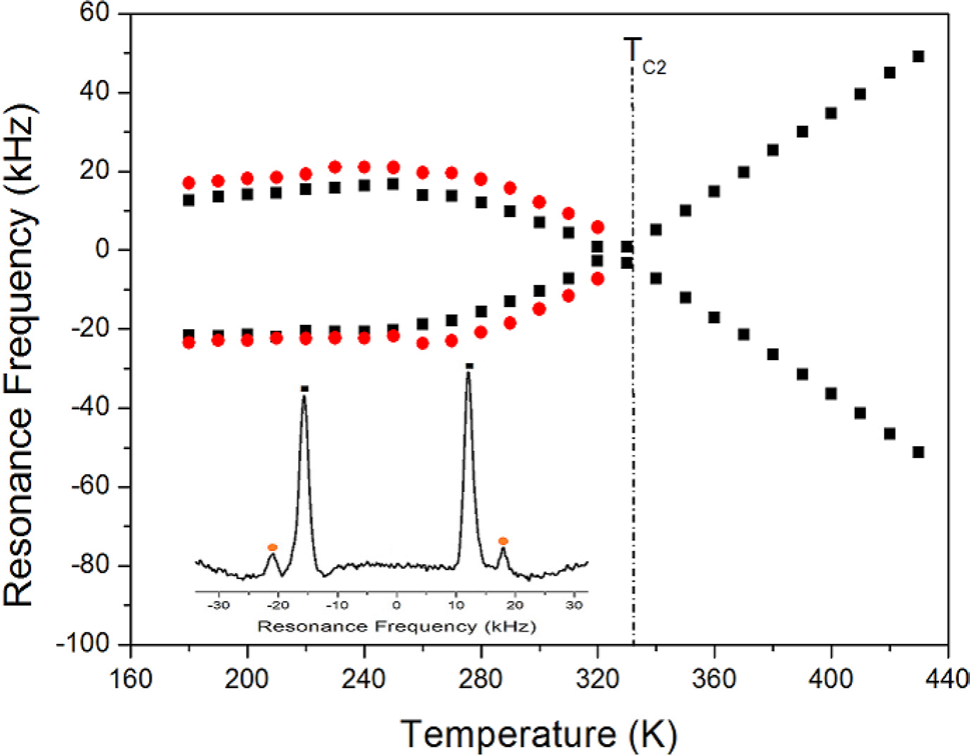
Temperature dependences on ^14^N resonance frequency of [(CH_2_)_3_(NH_3_)_2_] CuCl_4_ single crystal (Inset: static ^14^N NMR spectrum at 300 K).

^14^N NMR investigations were performed using a [(CH_2_)_3_(NH_3_)_2_]CuCl_4_ single crystal over the temperature range of 180–430 K. The ^14^N spectra were obtained using the solid-state echo method by static NMR at a Larmor frequency of 28.90 MHz. Two ^14^N NMR signals were derived from the quadrupole interactions due to the spin number I = 1. Near 333 K (= T_C2_), the number of resonance lines and resonance frequency of the NMR spectrum showed abruptly changes, as shown in Fig. 8. Above T_C2_, the spectrum showed one pair of lines, whereas below T_C2_ it showed two pairs. The lines with the same colour below T_C2_ indicate the same pairs for ^14^N. The changes in the ^14^N resonance frequency as a function of temperature were attributed to variations in the structural geometry, corresponding to changes in the quadrupole coupling constant^30,31^. The resonance frequency of the ^14^N signals below T_C2_ changed almost continuously, and those of the ^14^N signal above this temperature also varied abruptly. Near T_C2_, the electric field gradient tensors at N sites varied, reflecting changes in the atomic configuration around the nitrogen atom. Although the phase transition temperature at T_C2_ reported previously^20^ was not observed in our DSC experimental results, the ^14^N NMR spectrum showed changes near T_C2_. The phase transition at T_C2_ exists, and ^14^N in the NH_3_ groups plays an import role in this phase transition. In contrast, the two different ^14^N spectra below T_C2_ are thought to have two inequivalent N sites or be due to twin domains. However, according to the previously reported X-ray results^21^, there have been no reports of two different N sites, and twin domains have been reported^22^. Czapla et al.^22^ suggested that the ferroelastic domains observed in the orthorhombic phase could be connected to a prototype tetragonal phase. Here, the [(CH_2_)_3_(NH_3_)_2_]CuCl_4_ crystal existed in three crystallographic phases: monoclinic (*2/m*) above 434 K, tetragonal (*4/mmm*) between 334 and 434 K, and orthorhombic (*mmm*) below 334 K. For the transition from the *4/mmm* of the tetragonal phase to the *mmm* of the orthorhombic phase, the domain wall orientations were expressed as *x* = 0 and *y* = 0. According to Aizu^32^ and Sapriel^33^, the equations of the twin domain walls reflected the ferroelasticity of the *4/mmm*F*mmm*. Hence, our results are thought to support the mechanism of ferroelastic twin domains. As a result, the separation of two ^14^N NMR lines into four ^14^N NMR lines under T_C2_ was due to the ferroelastic twin domain structure.

## Conclusion

To investigate the physical properties of [(CH_2_)_3_(NH_3_)_2_]CuCl_4_ perovskite crystals, we performed DSC, TGA, optical polarizing microscopy, and NMR spectroscopy. The structural roles of the [(CH_2_)_3_(NH_3_)_2_]^+^ cation in [(CH_2_)_3_(NH_3_)_2_]CuCl_4_ crystals were investigated by ^1^H MAS NMR, ^13^C CP/MAS NMR, and ^14^N static NMR as a function of temperature. The changes in chemical shifts in the ^1^H and ^13^C NMR spectra indicated changes in crystallographic symmetry. The NMR chemical shifts were related to the local field at the location of the resonating nucleus in the crystals. The ^1^H NMR chemical shift for NH_3_ changed more significantly with temperature than that of CH_2_ because being H-bonded, the ^1^H NMR chemical shift of the NH_3_ moiety is much more sensitive to temperature fluctuations, and varies significantly due to the variation in H-bond length with temperature. The ^13^C NMR chemical shift for CH_2_-1 increased slowly with increasing temperature, without any anomalous change. However, the shift for CH_2_-2, moved significantly to lower values with increasing temperature compared to CH_2_-1. The ^13^C NMR chemical shifts of CH_2_-2 closer to the N–H···Cl bonds were higher those of CH_2_-1. In addition, the abrupt change in the resonance frequency of the ^14^N nuclei observed near T_C2_ was attributed to a ferroelastic phase transition. The previously reported phase transition at T_C2_^20^ was not observed in DSC, but the ^14^N NMR data clearly indicated changes in atomic configuration at this temperature. The NH_3_ groups are coordinated by N–H···Cl bonds; thus, atomic displacements with temperature in the environment of the ^14^N nuclei are correlated with CuCl_4_.

^1^H and ^13^C T_1ρ_ have lower values in the order of milliseconds because T_1ρ_ is inversely proportional to the square of the magnetic moment of paramagnetic ions. The T_1ρ_ values for CH_2_ and NH_3_ protons were almost independent of temperature, but the CH_2_ moiety located in the middle of the N–C–C–C–N bond undergoes tumbling motion according to the BPP theory. The increase in ^13^C T_1ρ_ at high temperatures may be simply due to the change in distance rather than the change in correlation time. More importantly, the total correlation time τ_C_ is dominated by the electric relaxation correlation time, rather than the rotational correlation time of the paramagnetic.

## Supplementary information

Supplementary figures.
