# Supplementary material for: Structural characterization, thermal properties, and molecular motions near the phase transition in hybrid perovskite [(CH2)3(NH3)2]CuCl4 crystals: 1H, 13C, and 14N nuclear magnetic resonance
Source: Sci Rep. 2020 Nov 30;10:20853. doi: 10.1038/s41598-020-77931-0 (PMC7705681; doi:10.1038/s41598-020-77931-0)
Supplement: Supplementary file 1 — Supplementary figures. [file 41598_2020_77931_MOESM1_ESM.docx]

**Structural characterization, thermal properties, and molecular motions near the phase transition in hybrid perovskite [(CH_2_)_3_(NH_3_)_2_]CuCl_4_ crystals: ^1^H, ^13^C, and ^14^N nuclear magnetic resonance**

**Ae Ran Lim**

Analytical Laboratory of Advanced Ferroelectric Crystals, Department of Science Eduction, Jeonju University, Jeonju 55069, Korea


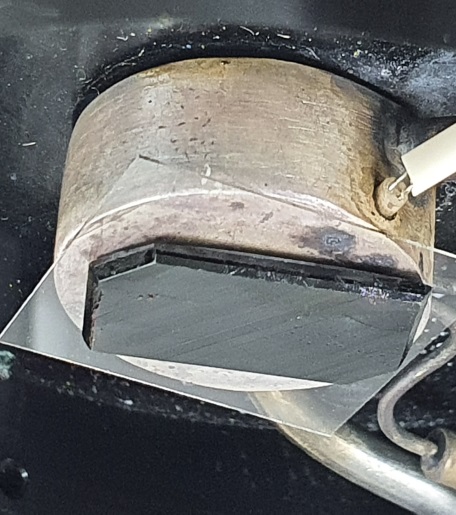

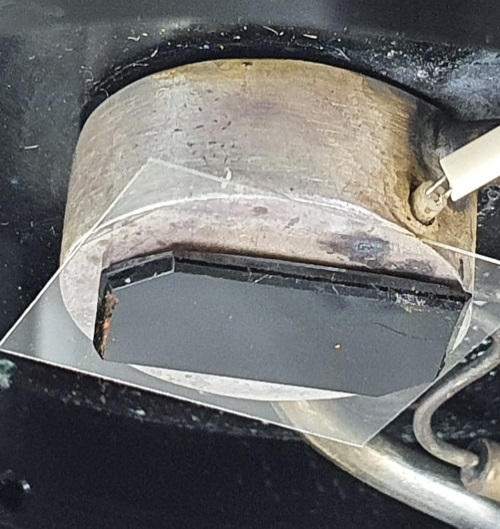

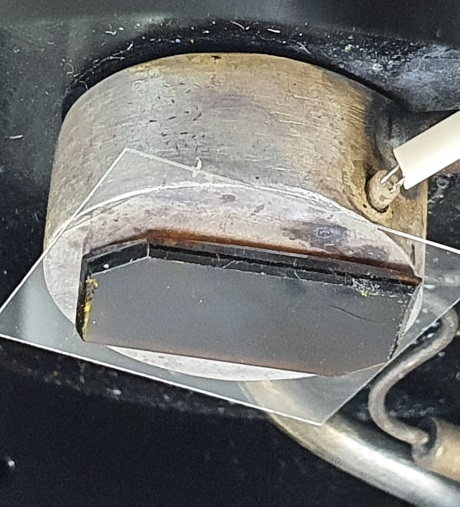


(a) (b) (c)


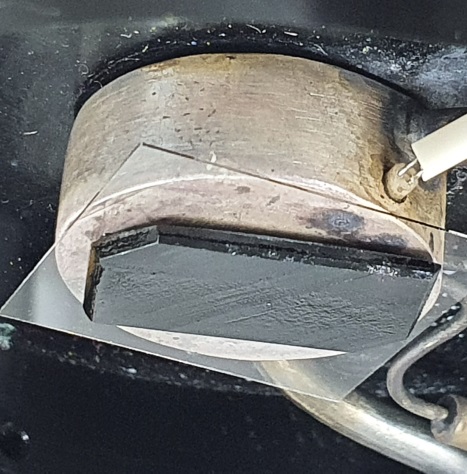

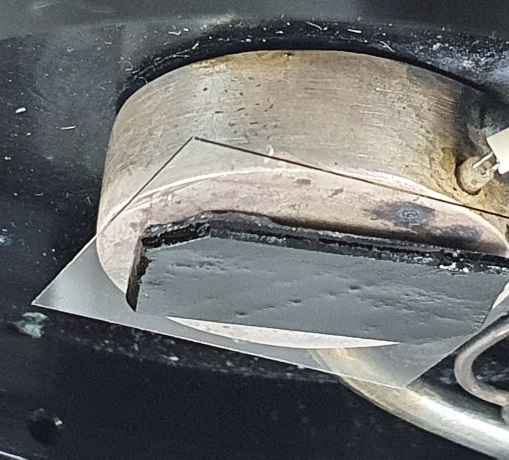

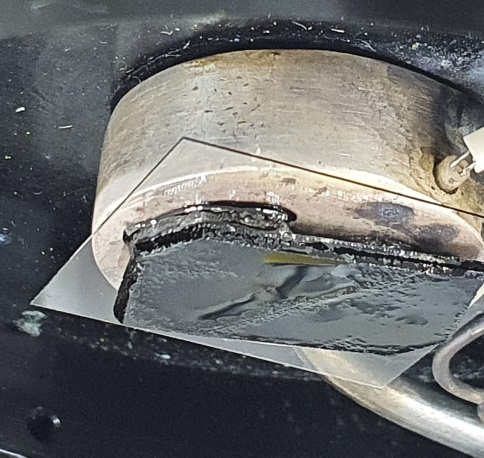


(d) (e) (f)

**Supplementary Information 1**. The states of the crystal at (a) 300 K, (b) 400 K, (c) 523 K, (d) 533 K, (e) 565 K, and (f) 603 K.


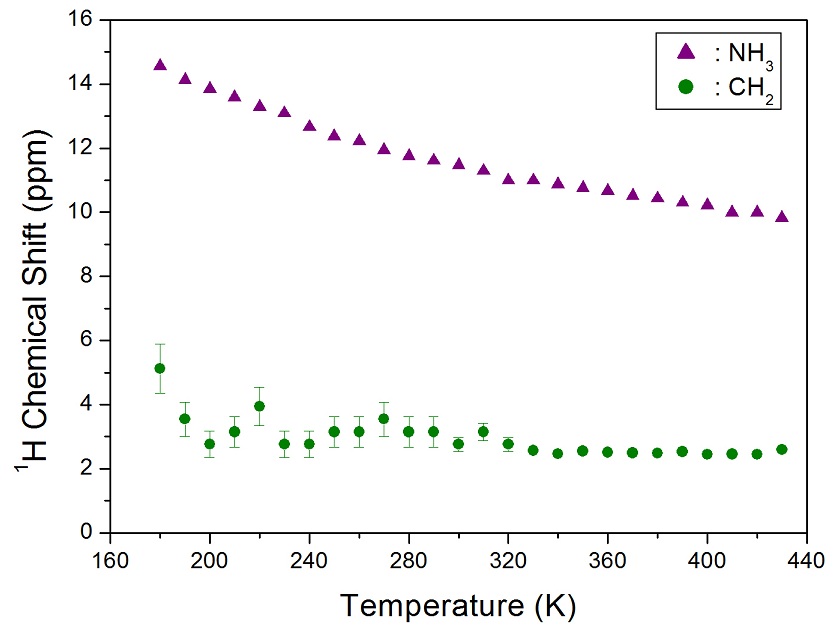


**Supplementary Information 2**. ^1^H NMR chemical shifts for CH_2_ and NH_3_ of [(CH_2_)_3_(NH_3_)_2_]CuCl_4_ as a function of temperatures.


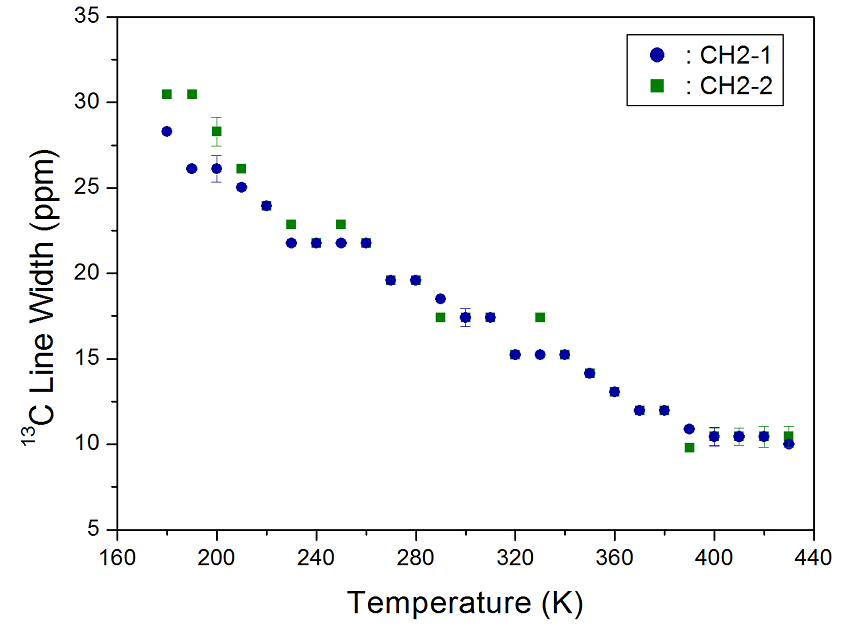


**Supplementary Information 3**. ^13^C NMR line width for CH_2_-1 and CH_2_-2 of [(CH_2_)_3_(NH_3_)_2_]CuCl_4_ as a function of temperatures.
